# Supplementary material for: Metasurface orbital angular momentum holography
Source: Nat Commun. 2019 Jul 19;10:2986. doi: 10.1038/s41467-019-11030-1 (PMC6642184; doi:10.1038/s41467-019-11030-1)
Supplement: Supplementary file 1 — Supplementary Information [file 41467_2019_11030_MOESM1_ESM.pdf]

# Supplementary Information

Ren, et al. “Metasurface orbital angular momentum holography”.

# Supplementary Information for

## Metasurface Orbital Angular Momentum Holography

Haoran Ren[1, 3*], Gauthier Briere[2], Xinyuan Fang[1], Peinan Ni[2], Rajath Sawant[2], Sébastien Héron[2], Sébastien Chenot[2], Stéphane Vézian[2], Benjamin Damilano[2], Virginie Brändli[2], Stefan A. Maier[3], and Patrice Genevet[2,*]

1) Laboratory of Artificial-Intelligence Nanophotonics, School of Science, RMIT University, Melbourne, Victoria 3001, Australia.

2) Université Côte d'Azur, CNRS, CRHEA, rue B. Gregory, 06560 Valbonne, France.

3) Chair in Hybrid Nanosystems, Nanoinstitute Munich, Faculty of Physics, Ludwig-Maximilians-University Munich, 80539 Munich, Germany.

Email: haoran.ren@physik.uni-muenchen.de; patrice.genevet@crhea.cnrs.fr.

## Supplementary Text

### Supplementary Note 1: OAM conservation in the holographic reconstruction process

According to Fourier integral theorem, the complex amplitude distribution of electric field in the hologram plane can be decomposed into an infinite number of plane-wave spatial frequency components as:

$$E(x,y)=\iint_{k_x,k_y<R} E_H(k_x,k_y)\exp[-i(k_x x+k_y y)]dk_x dk_y \tag{S1}$$

where $(x, y)$ and $(k_x, k_y)$ represent the orthogonal coordinates in the image plane and hologram plane, respectively. Equation S1 can be expressed as a Fourier transform of the field distribution in the hologram plane:

$$E(x,y)=\Im(E_H(k_x,k_y)) \tag{S2}$$

In the Fourier optics, the electric fields in the hologram plane and image plane form a Fourier pair, as such, the complex amplitude distribution of electric field in the image plane satisfies

$$E_I(x,y)=\Im(E_H(k_x,k_y)) \tag{S3}$$

which indicates that the spatial frequency distribution of a hologram physically equals to the complex amplitude distribution in the image plane in the Fourier transform holography, namely,

$$E(x,y)=E_I(x,y) \tag{S4}$$

When an incident beam carrying the OAM is used for the holographic reconstruction, the constituent spatial-frequency components of the hologram are further superposed with a helical phase wavefront:

$$E_H^{OAM}(k_x,k_y)=E_H(k_x,k_y)\bullet\exp(il\varphi) \tag{S5}$$

where $l$ is the topological charge of the OAM mode and $\varphi$ is the azimuthal angle in the polar coordinate system. Inserting Equation S5 into S2, we can obtain the spatial frequency distribution of the hologram based on an incident OAM beam:

$$E^{OAM}(x,y) = \Im(E_H^{OAM}(k_x,k_y)) = \Im(E_H(k_x,k_y)) * \Im(\exp(il\varphi)) \tag{S6}$$

where * denotes the convolution operation. Based on relationship in Equation S4, we can arrive at the relationship between the electric fields in the image plane and hologram plane based on an OAM incident beam:

$$E_I^{OAM}(x,y) = \Im(E_H(k_x,k_y)) * \Im(\exp(il\varphi)) = E_I(x,y) * \Im(\exp(il\varphi)) \tag{S7}$$

which indicates that the OAM-reconstructed electric field in the image plane is a convolution between the electric field of a holographic image and the Fourier transform of a helical wavefront. Mathematically, the Fourier transform of a helical wavefront, which acts as the kernel function of the convolution, is simply copied in each pixel of the holographic image.

Owing to the cylindrical symmetry of an optical beam, the Fourier transform of a complex helical wavefront can be calculated as (*53*)

$$E(\rho,\theta) = \Im(\exp(il\varphi)) = \frac{(-1)^{l+1}k}{f}\exp(il\varphi)\int_0^R J_l(\frac{k}{f}r\rho)rdr \tag{S8}$$

where $k$=2π/λ is the wavenumber of incident light and $f$ is the focal length of a Fourier lens, respectively. $r$ and $\rho$ are the radii in the hologram plane and image plane, respectively. The spatial frequency of a helical wavefront is represented by a doughnut-shaped intensity distribution in the image plane (Supplementary Figure 3).

Since the reconstruction is based on a convolution in the Fourier domain, the OAM property of the incident OAM beams are preserved in each pixel of the reconstructed holographic images (see Equation S7), if and only if the spatial sampling of the holographic

image $E_I(x, y)$ avoids spatial overlap of the helical wavefront kernel (see Equation S8). In Equation S8, the sampling period (*p*) is determined by the topological charge (*l*) of an OAM mode, the effective numerical aperture (NA=sin(atan(*R*/*f*))) of the hologram, and the wavenumber (*k*) of the incident light, respectively. In this paper, without the loss of generality, OAM-dependent sampling constants *(p*) were numerically characterized by calculating a hologram with an effective numerical aperture of 0.05 at a wavelength of 632 nm (Fig. 2A).

**Supplementary Note 2: Numerical design of an OAM-conserving meta-hologram**

Based on the OAM-dependent sampling constant (*p*) in Fig. 2A, an OAM-conserving meta-hologram can be designed. In this context, multiplying a desired image object with an OAM-dependent two-dimensional (2D) Dirac comb function in the image plane gives rise to an OAM-conserving hologram with a discrete spatial frequency distribution. As such, the image target becomes two-dimensional entity field which can be written as $\sum_{i=1}^{M} I_i^d \delta(x_i, y_i)$, where *M* denotes the total pixel number and $I_i^d$ denotes the desired peak intensity of *i*th pixel in the desired image. To numerically calculate the required phase distribution in an OAM-conserving meta-hologram, a two-dimensional Fourier transform based iterative phase retrieval method was used. To determine the pixel number in an experimentally fabricated meta-hologram with a physical size of 200 µm by 200 µm, we considered the subwavelength resolution (340 nm by 340 nm) of the meta-hologram, which results in the pixel number of 588 by 588. Setting the reconstruction distance of meta-holograms as 2 *mm* turns out an effective numerical aperture of 0.05 of meta-holograms. To maximize the diffraction efficiency on the holographic image, a 2D weighting factor *w* defined as $w^n = w^{n-1} \frac{\sum_{i=1}^{M} abs(E(x_i, y_i))}{M * abs(E(x_i, y_i))}$ ( $w^0 = 1$) is embedded in each phase-retrieval iteration to individually manipulate the intensity distribution in each pixel of

holographic images, where $n$ is the iteration number. Notably, to achieve the lensless reconstruction of a holographic image directly from a meta-hologram, the phase function of a Fourier transform holographic lens is further added on an OAM-conserving meta-hologram (Supplementary Figure 4) which was further digitized based on five different-sized GaN nanopillars. Accordingly, OAM-selective and -multiplexing meta-holograms have been designed, respectively.

**Supplementary Note 3: Numerical design of an OAM-multiplexing meta-hologram**

To design an OAM-multiplexing hologram, an intensity-weighting factor was added onto each OAM-selective hologram to obtain a uniform intensity distribution among the OAM-reconstructed holographic images. Specifically, a weighting factor $w_i^n$ defined as ( $w_i^n = w_i^{n-1} \frac{\sum_{i=1}^{4} abs(E(x_i, y_i))}{4 * abs(E(x_i, y_i))}$ ) was embedded in each iteration to iteratively optimize the intensity distribution of holographic images, where $i$=1, 2, 3, 4 and $n$ represent four selected pixels from the multiplexing holographic images and the iteration number, respectively. As a result, a phase-only OAM-multiplexing meta-hologram capable of offering a uniform intensity distribution among the reconstructed holographic images can be designed, based on the relationship of $\Phi^n = \arg(w_1^n e^{i2\pi\varphi_1} + w_2^n e^{i2\pi\varphi_2} + w_3^n e^{i2\pi\varphi_3} + w_4^n e^{i2\pi\varphi_4})$ , where $\varphi_1$, $\varphi_2$, $\varphi_3$, and $\varphi_4$ represent the four multiplexing holographic images, respectively.

**Supplementary Note 4: Experimental characterization of absolute efficiency of meta-holograms**

To characterize the efficiency of OAM metasurface holograms, we firstly measured the transmission efficiency of metasurfaces, $E_T$=$P_t$/$P_{in}$, at different visible wavelengths, where $P_t$ and $P_{in}$ represent the optical power transmitted from a meta-hologram and from a bare substrate

by moving the meta-hologram in and out of an incident laser beam, respectively. Secondly, we characterized the diffraction efficiency of meta-holograms, $E_D=P_{im}/P_t$, at different visible wavelengths, where $P_{im}$ and $P_t$ represent the amount of energy in a holographic image and the optical power transmitted from a meta-hologram, respectively. The measurement is realized by placing an aperture in an intermediate image plane in order to selectively monitor the optical power of the holographic image, thus removing the zero-order transmission. The absolute efficiency ($E$) of meta-holograms was calculated based on the relationship of $E=E_T*E_D$ and plotted in Supplementary Figure 13.

## Supplementary Note 5: Numerical characterization of effective mode index of GaN nanopillars

The resonant near-field patterns of the electric field inside the longitudinal plane of a GaN nanopillar with a height of 1 μm have been numerically characterized at a wavelength of 632 nm, which indicate a large phase advancement reaching 2π (insets in Supplementary Figure 14). The insets show the near-field electric field patterns of five selected nanopillars with radii of 76 nm, 80 nm, 86 nm, 94 nm, and 104 nm for the digitalization of our OAM meta-holograms. Considering the GaN nanopillar as a dielectric waveguide, we have performed the characterization of the effective mode index of nanopillars with a different radius (Supplementary Figure 14). The resultant large effective mode index change as a function of radius opens the possibility of a strong phase shift using GaN nanopillars, laying the physical foundation of the strong phase sensitivity to the radius of GaN nanopillars.

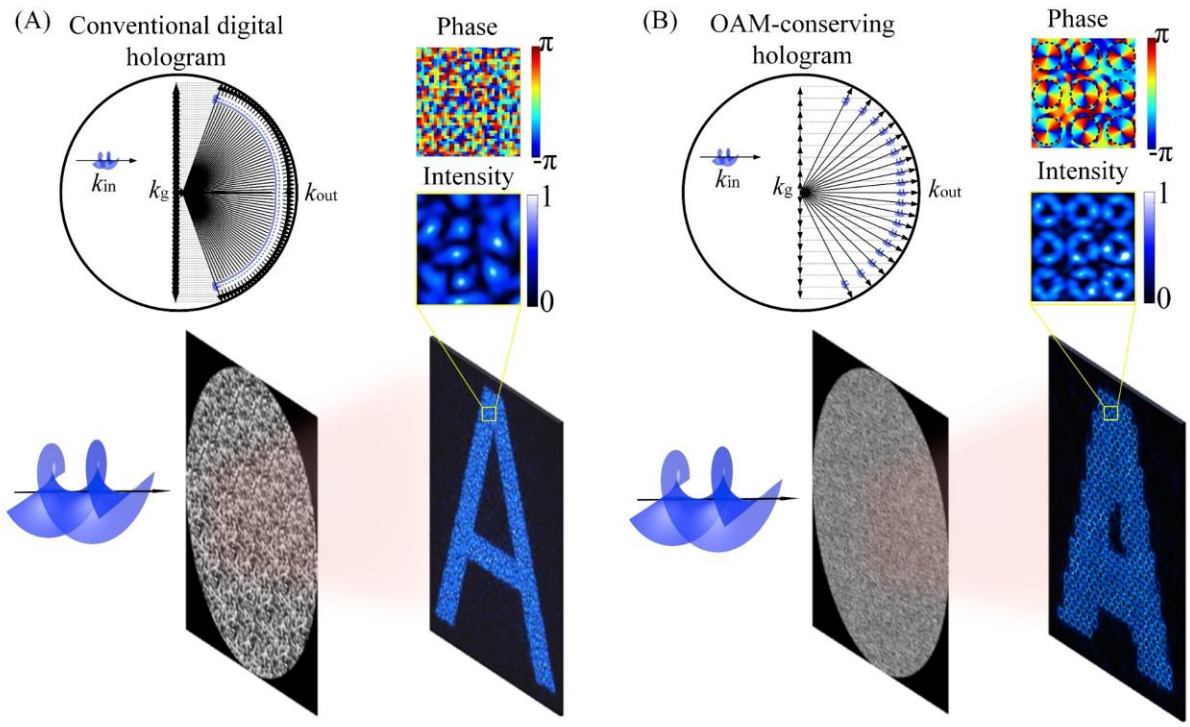


**Supplementary Figure 1. Comparison of a conventional digital hologram with an OAM-conserving hologram.** (**A**) A conventional digital hologram with a quasi-continuous spatial frequency distribution breaks the helical wavefront carried by an incident OAM beam. The inset (left) shows a quasi-continuous spatial-frequency content of the hologram. The insets (right) show the phase and intensity distributions of an enlarged pixel in an OAM-reconstructed holographic image, respectively. (**B**) An OAM-conserving hologram with a discrete spatial frequency distribution, capable of preserving a helical wavefront from an incident OAM beam. The inset (left) shows a discrete spatial frequency distribution of an OAM-conserving hologram. The insets (right) show the phase and intensity distributions of an enlarged pixel in an OAM-reconstructed holographic image, respectively.

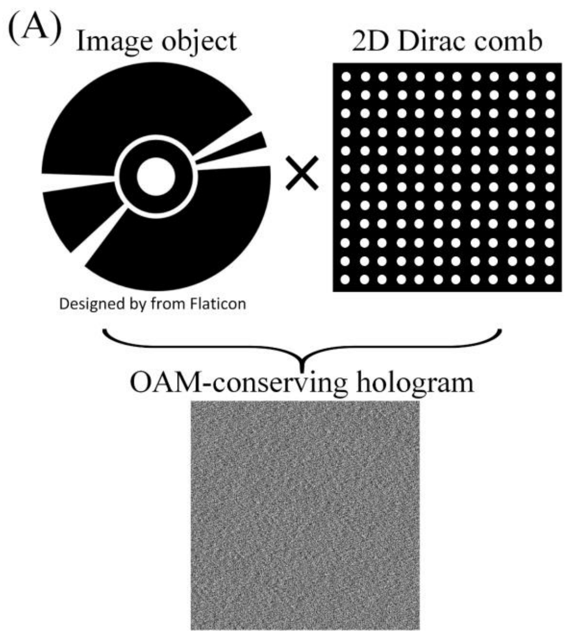


**Supplementary Figure 2.** Schematic design of OAM-conserving holograms through multiplying an object image with an OAM-dependent 2D Dirac comb function (a constant periodicity: $p$) in the image plane. The original "disc" and "car" images were obtained from Flaticon and Free Icons Library websites, respectively.

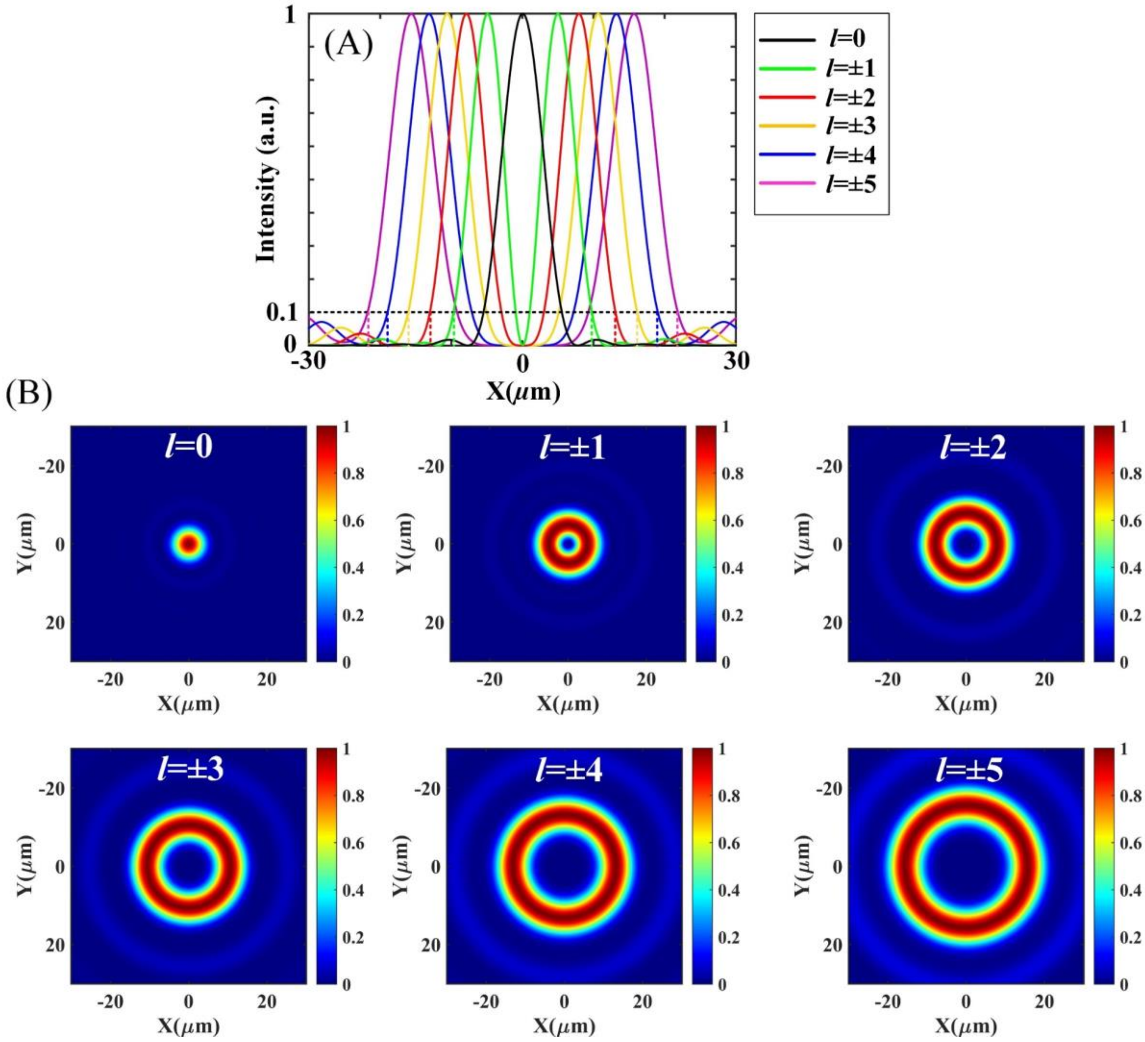


**Supplementary Figure 3. Numerical characterization of the spatial frequency of different incident OAM beams represented by spiral phase plates with various topological charges.** (**A**) The intensity cross-sections of spatial frequency distributions of different OAM modes. The dotted line labels out the positions used for defining the sampling constant (*p*) in Fig. 2A. (**B**) The intensity distributions of different incident OAM beams with various topological charges in the image plane.

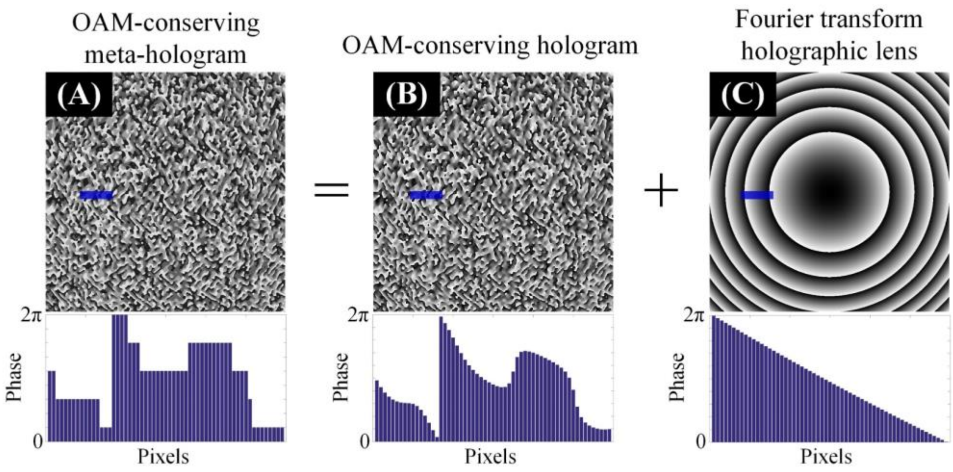


**Supplementary Figure 4. Design of an OAM-conserving meta-hologram (A) by superposing an OAM-conserving hologram (B) and a Fourier transform holographic lens (C).** Diagrams in the bottom show phase distributions along blue lines labelled out in (A), (B), and (C), respectively.

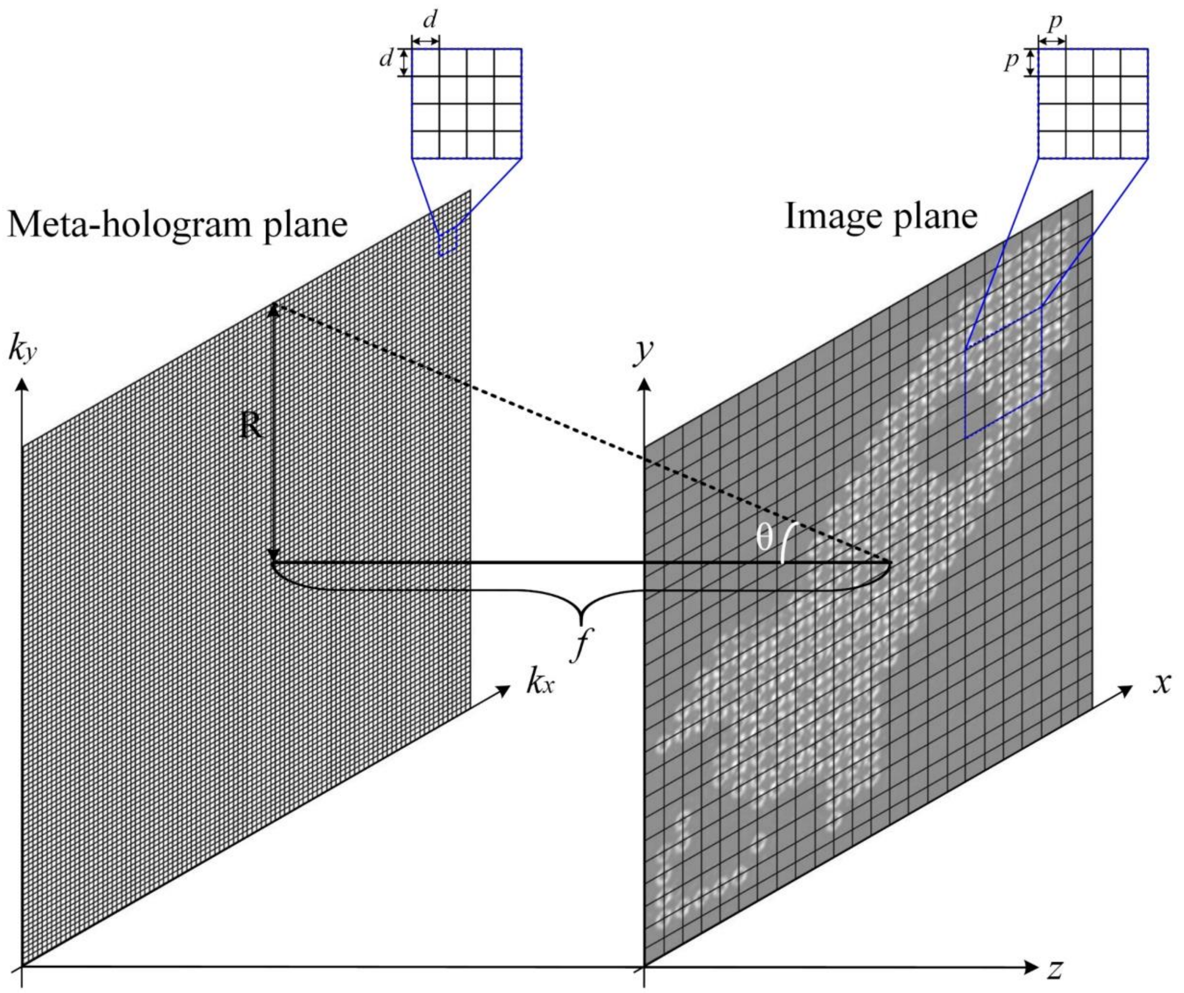


**Supplementary Figure 5. Schematic illustration of the lensless reconstruction of an OAM-carrying holographic image from an OAM-conserving meta-hologram.** $R$ and $f$ represent the radius and reconstruction distance of a meta-hologram, respectively. $\theta$ angle is used to calculate the effective numerical aperture of the meta-hologram.

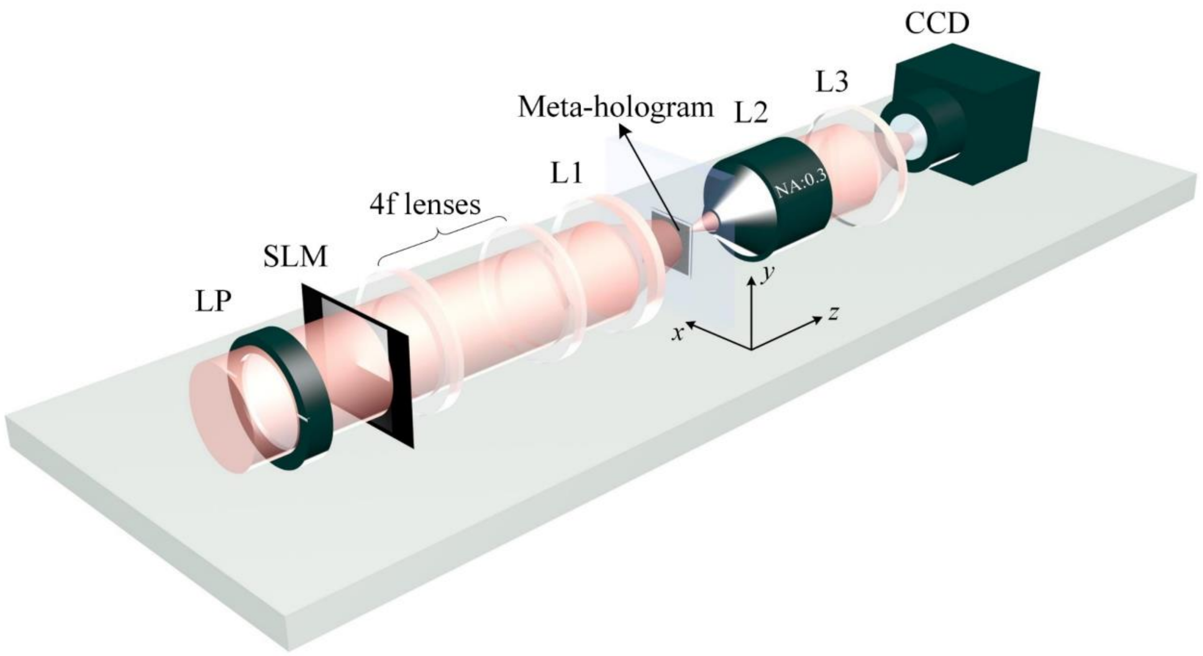


**Supplementary Figure 6. Optical setup for the experimental demonstration of metasurface OAM holography, where a spatial light modulator (SLM) is used to generate different incident OAM beams that are weakly focused by a lens (L1) down to OAM-conserving (-selective and -multiplexing) meta-holograms.** CCD: charge-coupled device.

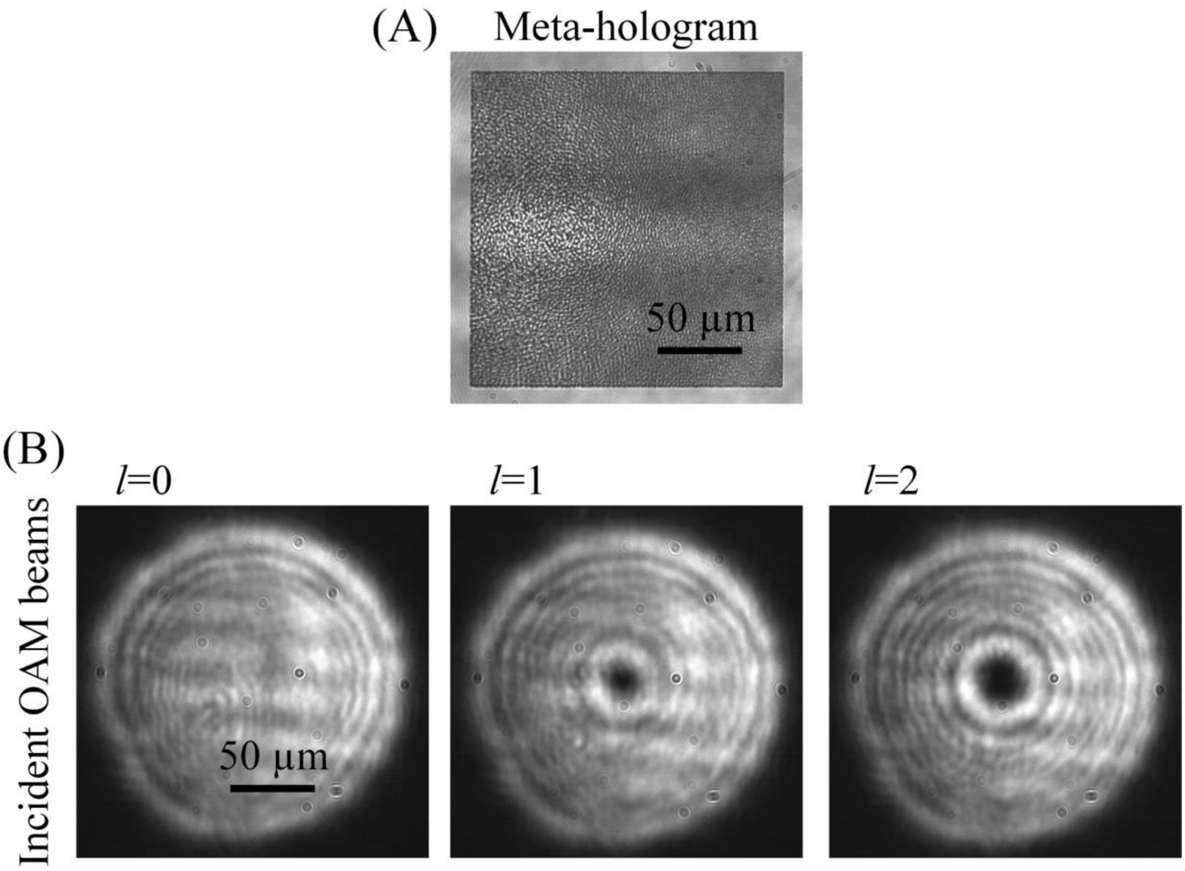


**Supplementary Figure 7. Microscopic optical images of an OAM-conserving meta-hologram (A) and the incident OAM beams with topological charges of *l*=0, 1, and 2 (B), respectively.**

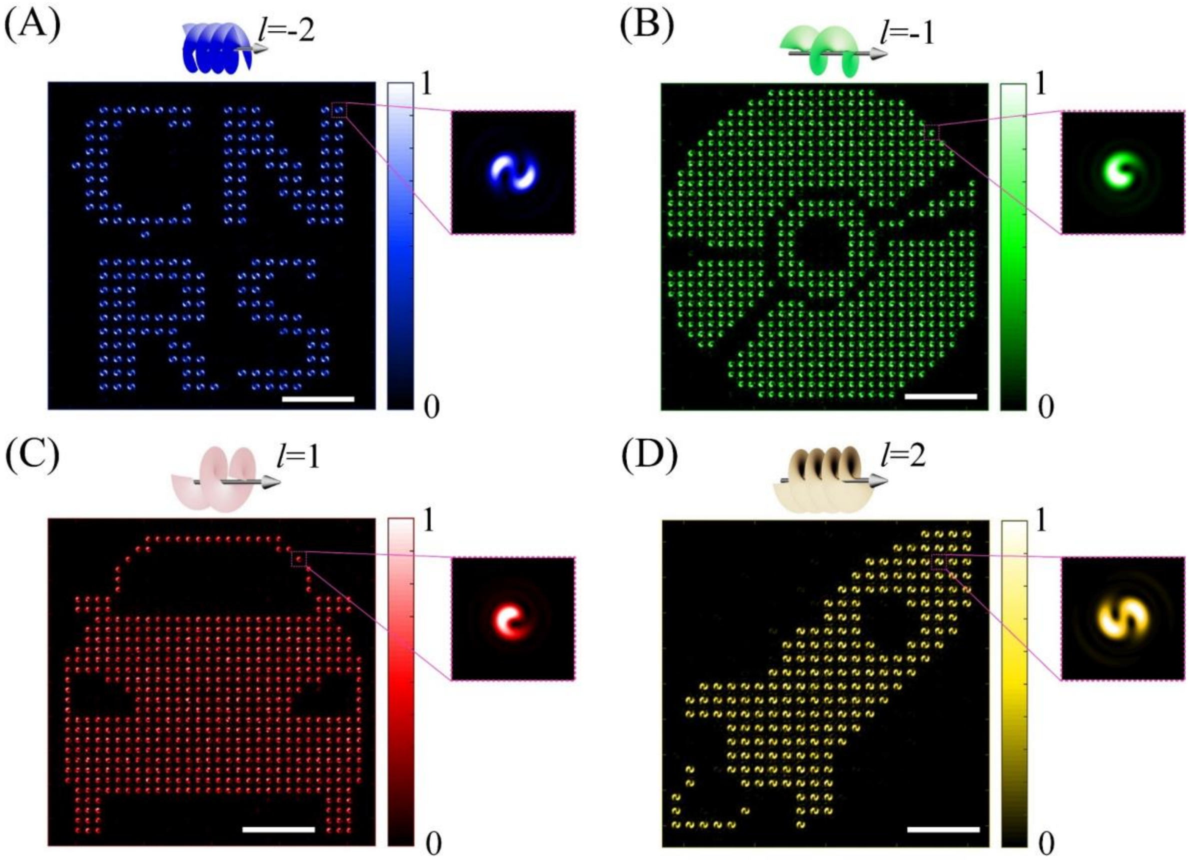


**Supplementary Figure 8. The interference of a spherical reference beam with OAM-reconstructed holographic images, with the use of different OAM modes with topological charges of *l*=-2 (A), -1 (B), 1 (C), and 2 (D), respectively.**

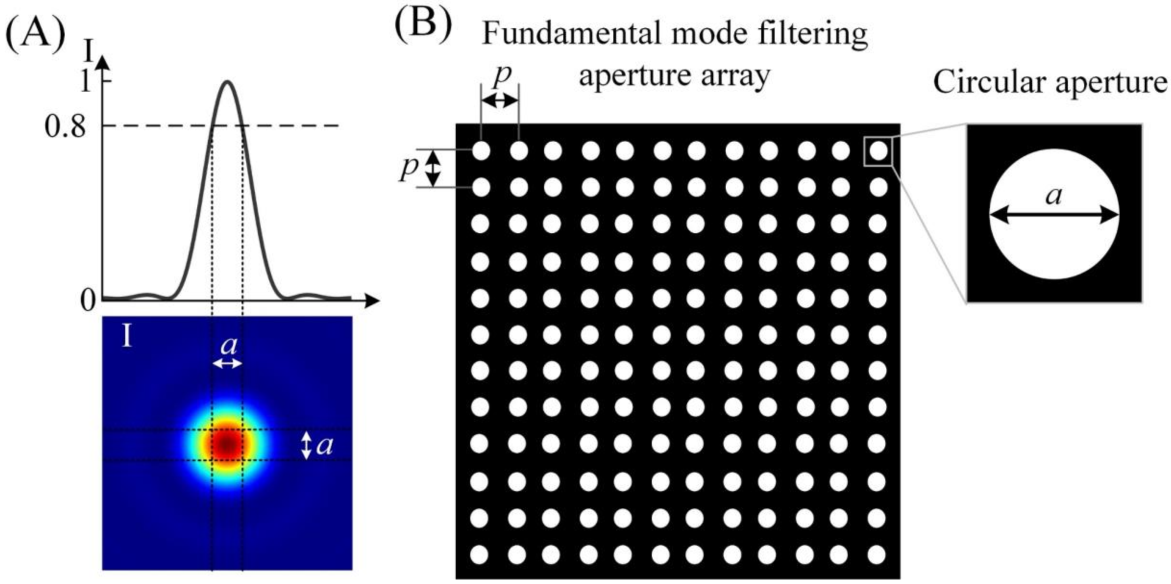


**Supplementary Figure 9. Implementation of a fundamental mode filtering aperture array in the detector plane to further improve the OAM selectivity by an OAM-selective meta-hologram.** (**A**) The intensity distribution of a fundamental spatial mode in the image plane, where *a* denotes the size of a circular aperture. (**B**) Schematic of a fundamental mode filtering aperture array, where *p* labels out the periodicity of the aperture array. Inset: an enlarged circular aperture with a diameter of *a*.

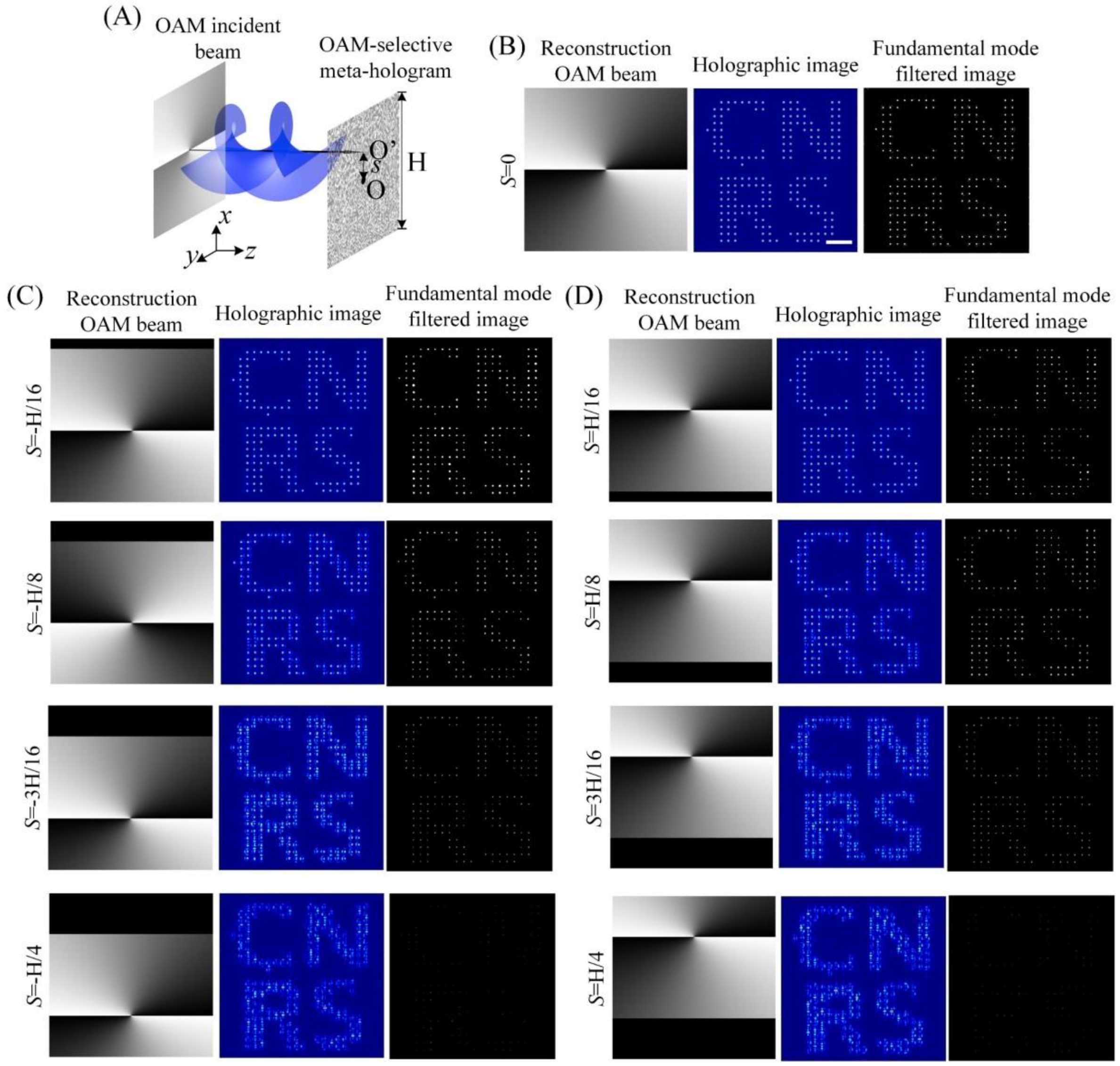


**Supplementary Figure 10. Numerical characterization of the impact of misalignment of an incident OAM beam with respect to an OAM-selective meta-hologram.** (**A**) Schematic illustration of the misalignment between and an incident OAM beam and an OAM-selective meta-hologram. (**B-D**) Holographic reconstruction based on an OAM-selective meta-hologram with a spatial shift with respect to an OAM reconstruction beam.

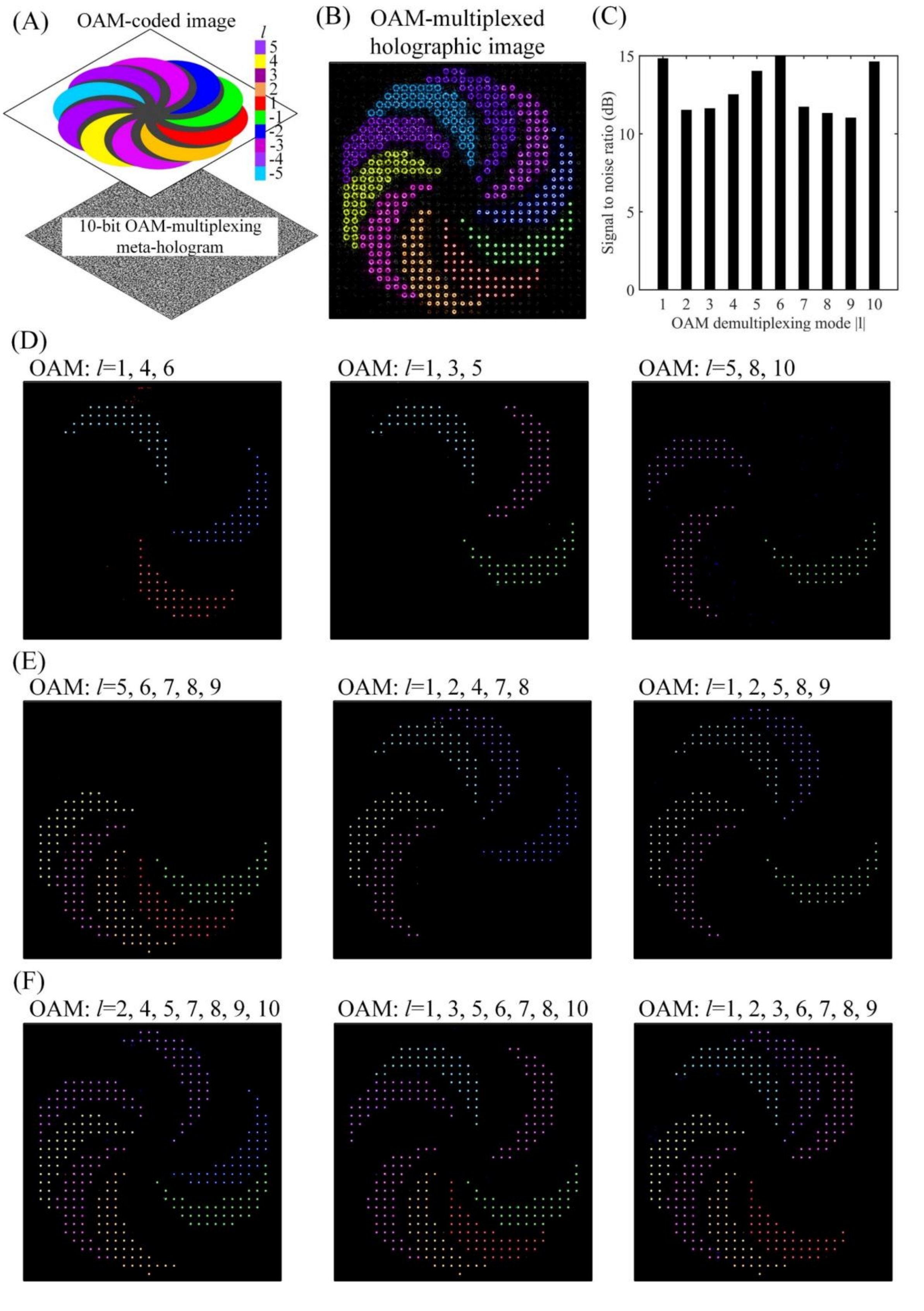


**Supplementary Figure 11. Design and numerical characterization of a 10-bit OAM-multiplexing meta-hologram capable of massive reconstruction of up to $2^{10}$ OAM-**

**dependent holographic images.** (**A**) Schematic of an OAM-coded image target and its resulting 10-bit OAM-multiplexing meta-hologram. Pseudo colors are used to visualize different OAM modes. (**B**) Reconstruction of an OAM-multiplexed holographic image based on a planar wavefront, leading to a holographic image carrying ten different OAM modes with topological charges spanning from -5 to 5. (**C**) Signal-to-noise ratio of different OAM modes in the holographic demultiplexing. (**D**-**F**) Exemplary holographic images reconstructed from the 10-bit OAM-multiplexing meta-hologram (9 out of $2^{10}$), with the use of three (D), five (E), and seven (F) incident OAM beams, respectively.

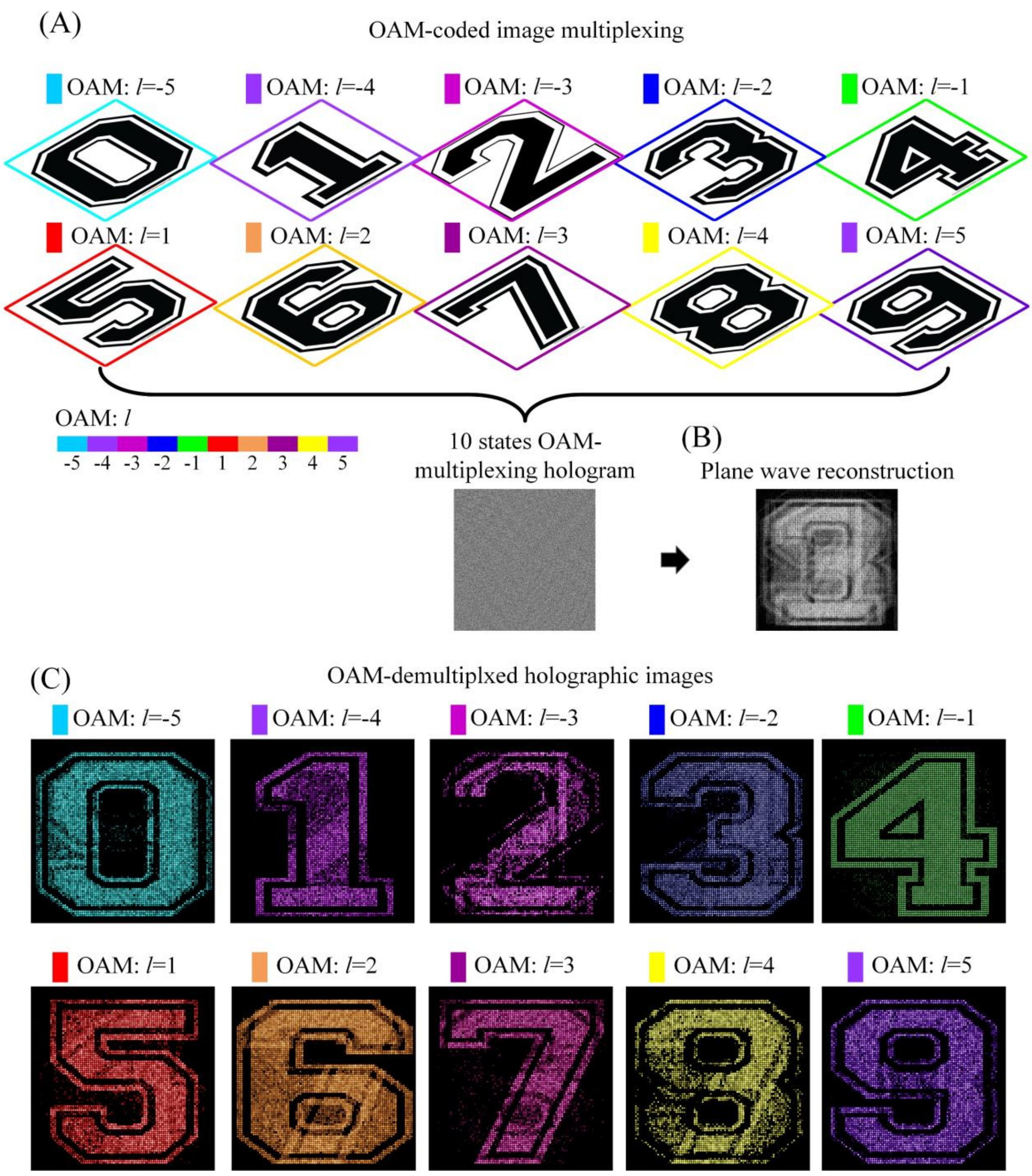


**Supplementary Figure 12. Numerical characterization of an OAM-multiplexing hologram capable of the lensless reconstruction of 10 distinctive OAM-dependent holographic images.** (**A**) OAM-encoded image multiplexing based on 10 OAM states with a topological charge ranging from -5 to 5. (**B**) The reconstruction of a complex interference pattern from the 10 states OAM-multiplexing hologram through an incident beam with a planar

wavefront. (**C**) Numerical reconstruction of ten distinctive OAM-dependent holographic images through incident OAM beams with a topological charge from -5 to 5.

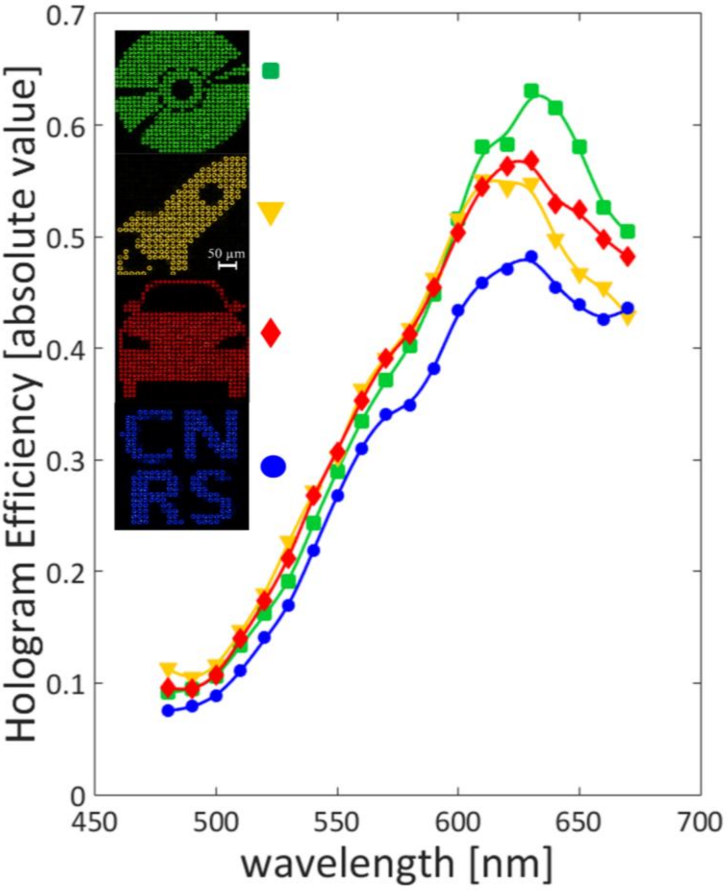


**Supplementary Figure 13. Experimentally characterized absolute efficiency of four different OAM metasurface holograms.**

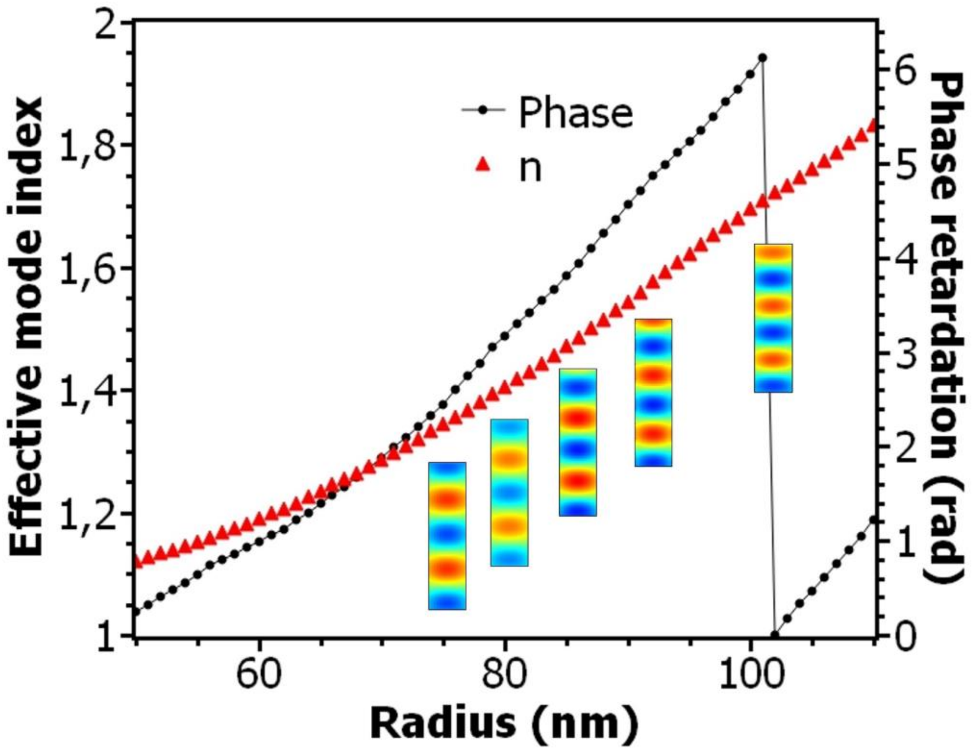


**Supplementary Figure 14. Numerical characterization of effective mode index and phase retardation of GaN nanopillars as function of the radius at a wavelength of 632 nm.** Insets show the electric field distributions in a longitudinal plane inside the GaN nanopillars with radii of 76 nm, 80 nm, 86 nm, 94 nm, and 104 nm, respectively.

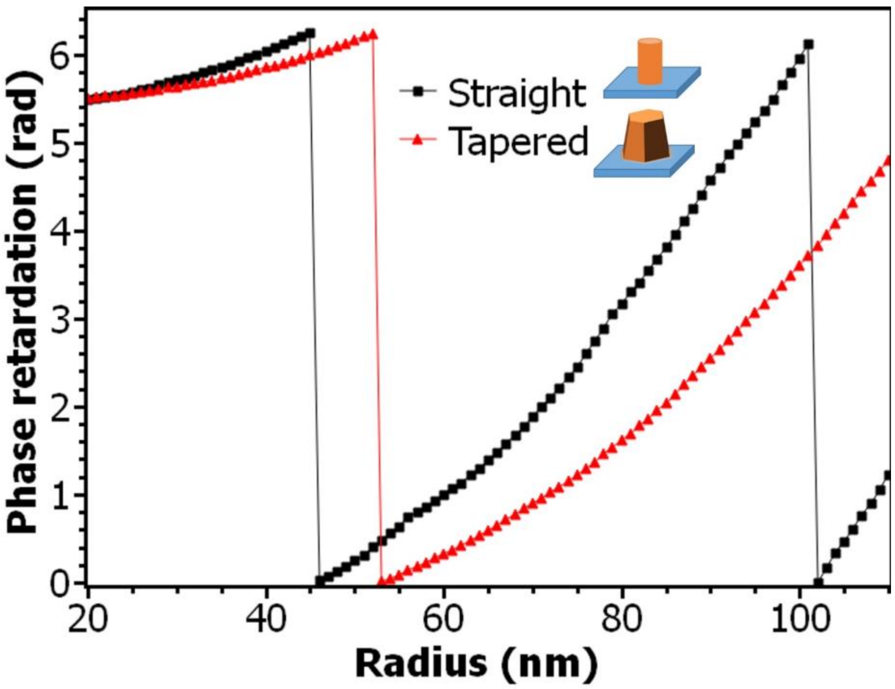


**Supplementary Figure 15. Numerical characterization of the influence of the tapering effect of GaN nanopillars on the phase retardation.**
